# ^18^F-FDG PET/CT Imaging of Primary Hepatic Neuroendocrine Tumor

**Published:** 2015

**Authors:** Katsuya Mitamura, Yuka Yamamoto, Kenichi Tanaka, Takayuki Sanomura, Makiko Murota, Yoshihiro Nishiyama

**Affiliations:** Department of Radiology, Faculty of Medicine, Kagawa University, Kagawa, Japan

**Keywords:** ^18^F-FDG, Neuroendocrine tumor, PET, Primary hepatic neuroendocrine tumor

## Abstract

Primary hepatic neuroendocrine tumors (PHNETs) are extremely rare neoplasms. Herein, we report a case of a 70- year-old man with a hepatic mass. The non-contrast computed tomography (CT) image showed a low-density mass, and dynamic CT images indicated the enhancement of the mass in the arterial phase and early washout in the late phase. F-18 fluorodeoxyglucose (^18^F-FDG) positron emission tomography (PET) and fused PET/CT images showed increased uptake in the hepatic mass. Whole-body ^18^F-FDG PET images showed no abnormal activity except for the liver lesion. Presence of an extrahepatic tumor was also ruled out by performing upper gastrointestinal endoscopy, total colonoscopy, and chest and abdominal CT. A posterior segmentectomy was performed, and histologic examination confirmed a neuroendocrine tumor (grade 1). The patient was followed up for about 2 years after the resection, and no extrahepatic lesions were radiologically found. Therefore, the patient was diagnosed with PHNET. To the best of our knowledge, no previous case of PHNET have been detected by ^18^F-FDG PET imaging.

## Introduction

Neuroendocrine tumors (NETs) are an uncommon type of cancer. Approximately 57% and 27% of NETs arise from the gastroenteropancreatic and bronchopulmonary systems, respectively (1). According to World Health Organization (WHO) categorization, gastroenteropancreatic NETs are classified as NET grade 1, NET grade 2, and neuroendocrine carcinoma (grade 3), which includes small-cell carcinoma and large-cell neuroendocrine carcinoma (2).

Primary hepatic neuroendocrine tumor (PHNET) is an extremely rare neoplasm. Presence of PHNET can be confirmed only when a primary extrahepatic source is excluded. Because it is so rare, staging and prognosis of PHNET are not fully determined (3). Until now, surgical resection has been regarded as the most effective treatment for PHNET, determining whether the tumors are operable at diagnosis (3).

Herein, we report a case of a patient with PHNET, undergoing F-18 fluorodeoxyglucose (^18^F-FDG) positron emission tomography (PET), with increased uptake in the hepatic mass.

## Case Report

A 70-year-old male patient was admitted to our hospital for a hepatic mass, which was identified incidentally during a routine health checkup. The patient was asymptomatic, and blood chemistry was normal. The CA19-9 serum level (74 U/mL; reference range: 0-37) was elevated, although carcinoembryonic antigen (CEA) and alpha fetoprotein (AFP) levels were normal.

**Figure 1:**
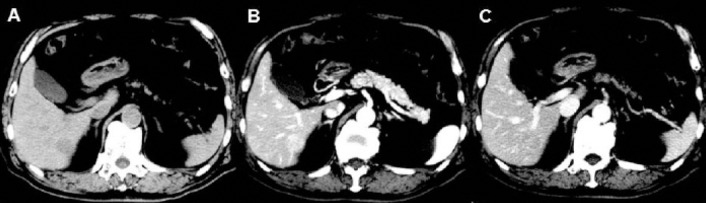
A non-contrast CT image shows a 23×18 mm, well-circumscribed, low-density mass in segment 6 of the liver (**A**). Dynamic CT images demonstrate the enhancement of the mass in the arterial phase (**B**) and early washout in the late phase (**C**)

**Figure 2:**
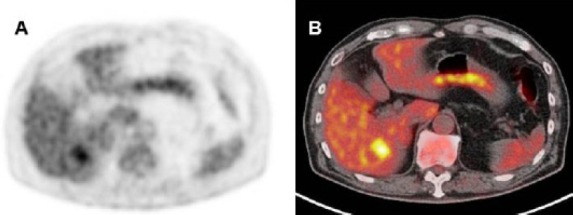
18F-FDG PET image shows an increased uptake in the liver, with a maximum standardized uptake value of 5.04 (**A**). Fused PET/CT image clearly shows that the accumulation corresponds to a liver mass (**B**)

Computed tomography (CT) images showed a 23×18 mm, well-circumscribed mass in segment 6 of the liver (Figure 1). ^18^F-FDG PET was performed using a Biograph mCT 64 slice PET/CT scanner. The dose of ^18^F-FDG was 370 MBq. ^18^F-FDG PET and fused PET/CT images showed increased uptake in the hepatic mass (Figure 2). Whole-body ^18^F-FDG PET images showed no abnormal activity except for the liver lesion.

**Figure 3:**
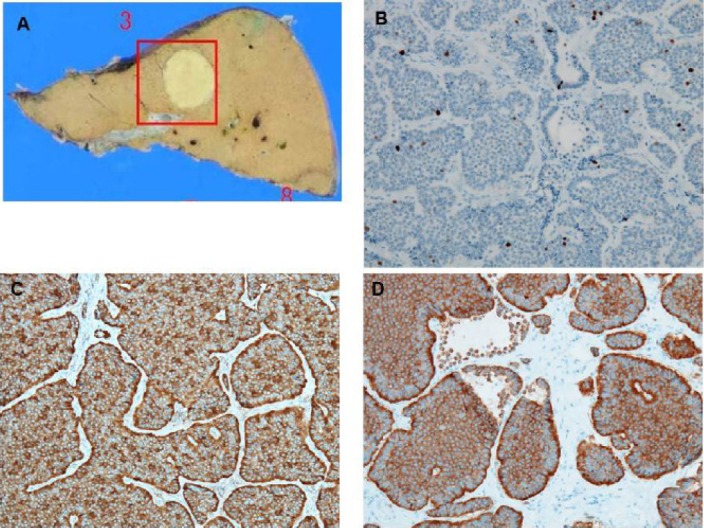
The image of the resected specimen shows a 22×20 mm solid mass (**A**). In Ki-67 immunohistochemistry, Ki-67 positive nuclei demonstrate a proliferation rate of 2% (**B**). Tumor cells proliferate in nests or trabeculae, presenting a focal rosette pattern. The tumor cells are immunoreactive for chromogranin A (**C**) and synaptophysin (**D**)

Presence of extrahepatic tumors was ruled out by performing upper gastrointestinal endoscopy, total colonoscopy, and chest and abdominal CT. A posterior segmentectomy was also performed. Histological and immunohistochemistry of the tumor confirmed a NET grade 1 (Figure 3).

The subject was followed up for about 2 years after the resection and no extrahepatic lesion was radiologically found. Therefore, the patient was diagnosed with PHNET.

## Discussion

NETs, also known as carcinoid tumors, are very rare and are usually found in the gastroenteropancreatic and bronchopulmonary systems (1). The liver is more often involved in metastatic lesions and occurrence of PHNET is extremely rare (4-6). The diagnosis of PHNET is usually confirmed through the exclusion of extrahepatic foci, which can be quite challenging.

It is quite difficult to distinguish PHNETs from other liver tumors, based on radiological findings. Somatostatin receptor scintigraphy is a helpful imaging procedure for the diagnosis of NETs, and its sensitivity has been estimated at up to 90% (7). Unfortunately, in the present case, somatostatin receptor scintigraphy was not performed due to its unavailability in our hospital.

Despite the high diagnostic sensitivity of somatostatin receptor scintigraphy, this modality provides limited information about the aggressiveness of NETs. Severi et al. investigated the role of ^18^F-FDG PET in patients with NET grade 1 and grade 2 (8). ^18^F-FDG PET was positive in 57% of patients with NET grade 1 and 66% of patients with NET grade 2 (8). Another study showed the high prognostic value of ^18^F-FDG PET for NETs, which exceeded the prognostic value of traditional markers such as Ki-67 proliferation index (9).

Although the diagnostic sensitivity of ^18^F-FDG PET is low for NETs, its prognostic value is high (10). To the best of our knowledge, no previous cases of PHNETs have been reported, using ^18^F-FDG PET imaging. Whole-body ^18^F-FDG PET scan can be also useful for excluding the extrahepatic sites of diseases.
